# Computer-Administered Screening of Reproductive-Aged Women for Diabetes Risk in Primary Care Settings, Feasibility and Acceptability of Such Screening, and Validity of Risk Assessments Based on Self-reported Weight

**Published:** 2007-06-15

**Authors:** Louise-Anne McNutt, Shazia Hussain, Martina Taylor, Eve Waltermaurer, Jeanne McCauley, Daniel E Ford, Jacquelyn C Campbell

**Affiliations:** State University of New York at Albany, School of Public Health, Department of Biostatistics and Epidemiology; State University of New York at Albany, School of Public Health, Department of Epidemiology and Biostatistics; State University of New York at Albany, School of Public Health, Department of Epidemiology and Biostatistics; State University of New York at New Paltz, Department of Sociology; The Johns Hopkins University School of Medicine and The Johns Hopkins University Bloomberg School of Public Health, Baltimore, Md; The Johns Hopkins University School of Medicine and The Johns Hopkins University Bloomberg School of Public Health, Baltimore, Md; The Johns Hopkins University School of Medicine and The Johns Hopkins University Bloomberg School of Public Health, Baltimore, Md

## Abstract

**Introduction:**

Obesity, a major public health problem, is the key modifiable component of diabetes risk. Addressing obesity and diabetes risk during primary care visits is recommended but, because of time constraints, is often difficult for health care providers to do. The purpose of this study was to determine whether technology can streamline risk assessment and leave more time to educate patients. We also tested the validity of self-reported weight in assessing diabetes risk.

**Methods:**

We recruited English-speaking women aged 18 to 44 years who came to a clinic for medical appointments from July through October 2003. Study participants completed a self-administered computer questionnaire that collected the following data: weight, height, family history of diabetes, level of exercise, amount of television time, and daily servings of fruits and vegetables. Self-reported and scale-measured weights were compared to determine the effect of self-reported weight on results of the American Diabetes Association's Diabetes Risk Test (DRT). In determining the sensitivity and specificity of self-reported weight, we used scale measurements as the standard.

**Results:**

Complete data were collected on 231 women, including 214 women without a history of a diabetes diagnosis. Compared with DRT results (determined by scale-measured weight), questionnaire results (determined by self-reported weight) had sensitivities of 93.9% (95% confidence interval [CI], 85.2%–97.6%) for high risk for diabetes and 90.4% (95% CI, 83.3%–94.7%) for moderate risk. The specificity of the self-administered DRT for any diabetes risk was 97.8% (95% CI, 88.4%–99.6%). About half the women reported discussing nutrition and exercise with their health care providers.

**Conclusion:**

Health care professionals can provide personalized diabetes education and counseling on the basis of information collected by self-administered computerized questionnaires. In general, patients provided a self-reported weight that did not substantially bias estimates of diabetes risk.

## Introduction

The U.S. Preventive Services Task Force (USPSTF) recently recommended obesity screening and weight loss counseling in primary care settings (1), an important response to the obesity and diabetes epidemics. Unfortunately, clinicians already face daily challenges to executing important preventive service recommendations within the time constraints of primary care (2). We hypothesized that technology can help streamline risk assessment and thus increase clinicians' opportunity for educating patients about their personal risk factors. Our study assessed the feasibility and acceptability of a diabetes-risk questionnaire administered to patients by computer touch screen while they were waiting to see a medical practitioner. A secondary purpose was to determine the validity of self-reported weight in assessing diabetes risk.

There are several ways to measure diabetes risk. At the urban clinic where we did our study, the physicians were familiar with and interested in administering the American Diabetes Association's (ADA's) Diabetes Risk Test (DRT) (3). Preventing or controlling diabetes is a priority for this community health center, which serves a predominantly African American urban community (4,5). The DRT is based on body mass index (BMI), age, family history of diabetes, and level of physical activity (3). It is valid when weight is measured using devices such as electronic scales, spring scales, and balance beam scales (2,6). The validity of DRT results based on self-reported weight immediately before a medical visit has not been assessed. In addition to using the DRT to assess risk for diabetes, some physicians use the results to educate patients about diabetes (7,8). However, DRT's feasibility as part of a large computer-administered health assessment has not been evaluated.

## Methods

Our study was conducted in a federally funded community health center serving a predominantly African American urban community. We recruited English-speaking women aged 18 to 44 years with a primary care appointment from July through October 2003. For this analysis, pregnant women were excluded because we could not assess their nonpregnant weight. The table on which the computers were set up was between the registration area and the waiting room, which allowed all eligible and willing patients to be screened and to participate in our study. All participants were asked to complete a brief touch-screen risk-assessment questionnaire before seeing their health care providers. A summary of each patient's responses to the questionnaire was printed and given to her to take to her provider, or the summary was given directly to the provider at the patient's request. Participants also received educational brochures on all topics covered in the questionnaire. A pen-and-paper questionnaire was also administered to obtain additional information on factors such as smoking, heart disease, and mental health. Because of confusion about the requirements of the newly implemented Health Insurance Portability and Accountability Act (HIPAA), we were not given patient lists from which to recruit participants. Hence, we were not able to calculate the response rate. The study was approved by the institutional review board at the State University of New York at Albany and the health center's patient board.

### Factors Measured 

#### Weight and height

Each participant self-reported height and weight using the computerized screening questionnaire: weight in pounds and height in feet and inches. Later, a nurse weighed each participant using a calibrated scale.

#### Body mass index

The computerized screening program was set to calculate BMI on the basis of the patient's height and weight. BMI is classified as follows: underweight (<18.5), normal weight (18.5–24.9), overweight (25.0–29.9), obese (30–39.9) and severely obese (≥40) (9). BMI was calculated twice using scale-measured weight and self-reported height. The English formula was used to calculate each participant's BMI (i.e., BMI = [weight in pounds/height in inches]^2^ x 703).

#### Diabetes risk

Diabetes risk was measured by a computer-administered version of the DRT (3). DRT scores of 0–2 indicate *very low risk* for diabetes; 3–9, *low to medium risk*; and ≥10, *high risk*.

#### Other risk factors for diabetes

The computerized screening questionnaire included questions regarding behaviors associated with risk for obesity and diabetes. These included number of days on which participants exercised per week, number of servings of fruits and vegetables consumed per day, and hours of television watched per day. The participants were asked how many days per week they exercised (defined, at a minimum, as walking fast for 20 minutes). We allowed drinking fruit or vegetable juice to be counted once a day as a serving of fruit or vegetables.

#### Feasibility and acceptability of diabetes risk screening

Physicians and nurses at the clinic were asked whether the computerized screen impeded patient flow (feasibility) and whether they were open to future computerized health screening (acceptability). To assess acceptability from the patients' perspectives, patients were asked "Is it acceptable to you to answer questions about your health on a computer?" and "How willing would you be to use a computer again to answer health questions?" In addition, a brief exit questionnaire asked patients whether they discussed exercise, nutrition, or other health behaviors during their visit with the health care provider.

### Data management 

Screening data were entered into a Microsoft Access database directly from the touch screen program. Survey data and measured weights were double entered and verified using EPI INFO (version 6, Centers for Disease Control and Prevention, Atlanta, GA) Data management and statistical analyses were conducted with SAS (version 9.1, SAS Institute, Cary, NC). Descriptive statistics, including frequencies and percentages, were computed. Self-reported and scale-measured weights were compared to determine the effect of self-reported weight on DRT risk assessment. To determine the sensitivity and specificity of the self-reported weight, the standard used was the scale-measured weight. Statistical associations were measured by chi-square tests for association.

## Results

Of the 268 women who were not pregnant and who agreed to participate in the study, 231 completed the screening questionnaire and survey sufficiently well for their data to be included in these analyses (self-reported weight was missing for 3 women, and scale-measured weight was missing for 34 women). Of the 231 whose data were used for the study, 214 reported no previous diagnosis of diabetes. Table 1 summarizes the demographic characteristics of the study population. Most women were African American, younger than 35 years of age, and had a high school education or higher. Fewer than 10% were college graduates.

### Demographic characteristics and diabetes risk 

Of the 231 study subjects, only 47 (20.3%) had DRT scores in the lowest risk category (0–2). Given their risk profiles, 144 (83.7%) of the 172 African American and Latina participants are at high risk for diabetes, will be at high risk for diabetes by age 45, or have diabetes. The percentage of white women in the lowest risk group (DRT score of 0–2) was higher than the percentages of black or Hispanic women in that group. For each age group, the most common risk category was *low risk* (DRT scores of 3–9). However, of the 111 women in that category, 110 (all except one) had scores of 5–9, which we categorized as "currently low risk, likely high risk when aged 45 years or older if risk factors do not change." Diabetes and diabetes risk increased with age as expected; women aged 34 to 44 years tended to be at higher risk than the younger women. Diabetes and diabetes risk were also associated with education level: the higher the level of education, the lower the risk for diabetes tended to be.

### Weight 

Scale-measured weight indicated 73.2% of women were above normal weight: 27.3% were overweight, 36.4% were obese, and 9.5% were severely obese. Of the 231 participants, 17 (7.4%) reported having a past diabetes diagnosis; of these, 15 were overweight, obese, or severely obese.

Approximately 16% of the women self-reported their weight accurately (i.e., their report was the same as their scale-measured weight). Study subjects were more likely to understate than overstate their weight: nearly 27% understated their weight by 5 or more pounds (Figure). Obese women were most likely to understate weight; however, they did not understate their weight enough to be categorized as having a healthy weight (Table 2).

**Figure 1:**
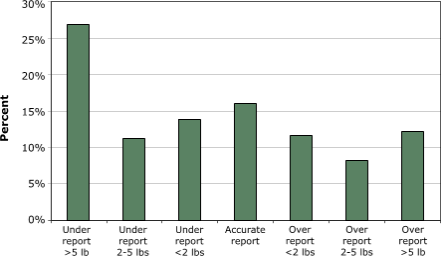
Accuracy of self-reported weight ascertained by scale-measured weight.

**Table:** 

Although understating weight was common, it had limited effect on DRT scores (Table 3). Among women reporting no prior diabetes diagnosis, the DRT based on self-reported weight had 93.9% (95% CI, 85.2%–97.6%) sensitivity for high risk and 90.4% (95% CI, 83.3%–94.7%) sensitivity for DRT scores of 5–9, which we classified as "currently low risk, likely high risk when aged 45 years or older if risk factors do not change." The specificity of self-reported weight was 97.8% (95% CI, 88.4%–99.6%), indicating that the DRT based on self-reported weight had limited effect on the risk classification of women with the lowest level of diabetes risk.

### Health behaviors and risk for diabetes 

The Centers for Disease Control and Prevention (CDC) recommends exercising 5 or more days a week, watching 1 hour or less of television per day and eating five or more servings of fruits and vegetables per day (10). Of the 231 women in our study, 68 (29.4%) reported that they exercised 5 or more days a week. Only 34 (14.8%) reported meeting the recommendation for television watching, and only 19 (8.2%) reported meeting the recommendation for fruit and vegetable consumption (Table 1).

Because exercise is a component of the DRT, we found a direct association between level of exercise and level of diabetes risk. Nutrition followed the expected pattern: the fewer daily servings of fruit and vegetables reported, the higher the DRT scores. Similarly, the less television watched per day, the lower the individual's risk score (Table 1).

### Feasibility, acceptability, and follow-up of the health risk questionnaire 

More than 90% of women found the computerized health questionnaire acceptable at some level and would be willing to complete similar questionnaires in the future. Acceptability was lowest for women who scored in the high-risk category for diabetes or who reported having had a diagnosis of diabetes (Table 4).

Among nurses and physicians in the clinic, 19 completed a brief survey on acceptability and feasibility. No staff member reported that computerized health screening interfered with patient flow, and 18 preferred computerized screening to screening by interview.

About half the study women reported discussing exercise or nutrition with their health care provider. No clear pattern of counseling about these behaviors was found by diabetes risk category. In general, the women were pleased with the health care provider's decision with regard to counseling, whether counseling was provided or not (Table 4).

In collaboration with the health educators at the clinic, we produced a report that was well received by both the clinic's health care practitioners and its patient board. In addition, 117 (50.6%) of the participants asked for the study report.

## Discussion

The utility of self-administered, computerized screening questionnaires based on USPSTF recommendations for screening and counseling are being assessed in medical settings. Computerized screening in the waiting room preserves time for health care practitioners to focus on personalized counseling in the examination room. Because computerized screening for diabetes risk is based on self-reported weight, it is vital to know whether self-reported weight would compromise the validity of DRT results. We found that, although only 83% of the women's BMIs were correctly classified through self-reports, the risk scores of 93% of women were correctly classified through the DRT. Only 6 (2.6%) of the 231 study women at risk for diabetes were not identified because of incorrect self-reported weight. In addition, the overall specificity was 97.8%.

Because most participants expected to be weighed by a nurse, we assumed that self-reported weight would be reasonably accurate. This was not the case. Our findings were similar to those of other studies of self-reported height and weight validity, which found that people with a high BMI are more likely than those of normal weight to underestimate their weight regardless of sex, age, race, or ethnicity (7,11-14). Few studies have been done to understand why this bias exists, particularly for overweight females. A cluster of surveys found that overweight people tend to feel greater discomfort with primary care providers than do normal-weight or underweight people (15-19). According to Lawlor and colleagues, another explanation is that overweight women are less likely to weigh themselves, thereby limiting the accuracy of their self-reports (13). Some studies (20,21) found that physicians and nurses are hesitant to weigh women during the medical visit because of the embarrassment and discomfort caused to the patient. And indeed, the physicians in our study specifically asked that weight not be printed on the patient summary. Nevertheless, it is important for health care providers to strike a balance between preserving patients' comfort now and preserving their health for the future by taking the opportunity to counsel them on weight control.

In addition to maintaining a healthy weight, people with type 2 diabetes can control their disease and those without the disease can reduce their risk of getting it through proper diet and exercise. Surprisingly, the study women who reported exercising at least five times per week were more likely than those who do not exercise enough to have risk scores of 5–9, which we categorized as "currently low risk, likely high risk at age 45 years if risk factors do not change."  These women may either exercise frequently for weight control or overestimate their amount of exercise.

Among the African American women in our study, 11.6% reported a previous diagnosis of diabetes, 30.0% were already at high risk for diabetes, and 46.4% will be at high risk by age 45 if no behavior or weight changes are made. Although obesity is one of the leading causes of mortality in the United States, it is a particularly important cause among African American women (22). In 2000, the prevalence of obesity in the United States was higher among African American women (49.7%) than among white women (30.1%) or Hispanic women (39.7%) (23). During the past decade, obesity increased more among African American women (11.5%) than among white women (4.4%) or Hispanic women (7.2%) (23).

Because obesity is increasing rapidly, there is an unprecedented focus on reducing its prevalence in order to achieve concurrent reductions in diabetes and heart disease. From a public health perspective, primary prevention needs to begin with young, at-risk people. The ADA DRT is a valid measure of current diabetes risk (3). However, it is not designed to educate young people who are at risk for diabetes. To address this issue, we divided the DRT *low risk* classification (3–9) into two: 3–4 indicates *low risk* and 5–9 indicates *currently low risk, likely high risk when 45 or older* for women who will be in the high risk group when they are 45 if they do not change their lifestyle. The purpose was to send a brief educational message to those at future risk because diabetes prevention hinges on early education and behavior modification.

These findings have certain limitations. Some women approached for this study refused to participate. Because of the health center's interpretation of HIPAA regulations, we were not permitted access to denominator information; without knowing the number of potential patients, we could not determine the response rate. In addition, we do not have information on women who refused to participate to determine how they may be similar to or different from women who participated. The substantial risk for diabetes for those in our sample is more than the risk for the general population of urban women (23), probably because the study was conducted in a health center where we recruited women consecutively according to their medical appointments. Therefore, the women in our study are likely to have more health problems than women in the general population.

Since height was not measured instrumentally, we used the self-reported height to calculate BMI with both self-reported weight and scale-measured weight. Research suggests that overweight and obese women are more likely than normal-weight women to overestimate height (7). This overestimate is typically small, but can affect estimated BMI and potentially the DRT score. We subtracted first 1 inch and then 2 inches from each woman's height to assess the potential effect of such a bias. The BMI categories and diabetes risk levels were not substantially affected. Bias would not be apparent until everyone overestimated height by 2 inches or more (data not shown). No other components of the DRT were validated in this study. Once screening began, women were asked to participate if they had a medical appointment. This was an arbitrary time in women's medical care, and therefore some providers may not have discussed issues related to diabetes risk because they had discussed the risks recently or the patient's current medical problem precluded such a discussion. Lastly, we used the ADA's DRT to screen women and educate them about diabetes risk. Although other instruments for screening (24) are available, we weighted heavily the request of the health center's physicians that we use the DRT.

Recently, Romera et al discussed concerns related to BMI measurements (25). In their article, they point out that BMI cannot differentiate between body fat and lean mass, thus leading to misclassifications of obesity. However, in our sample of urban women at a community health center, a high BMI most commonly indicates obesity, not the high level of fitness needed to obtain substantial muscle weight.

Changes in the U.S. population can be expected to affect the number of people with diabetes. By 2050, 14% of the population will be African American, and 24% will be Hispanic (26), two groups with a high prevalence of diabetes. Because the number of women at highest risk for diabetes and associated complications is increasing and because obesity and physical inactivity are on the rise, the number of people with diabetes is expected to continue to increase into the 21st century (27-30). 

Physicians should screen and counsel patients about obesity (2) and diabetes risk. Developing methods for effectively addressing obesity in primary care remains a challenge. Using a computerized screening program has several advantages over practitioner inquiry. The approach can save time for practitioners who provide preventive services. Many more questions can be asked by computer than a health care provider has time to ask, and the information gathered can be summarized immediately for both patients and clinicians. Physicians and other health care providers can use this information to focus on personalized counseling instead of risk assessment. For example, patients in our study got brochures about the health behaviors covered in the screening questionnaire as well as a printout of their screening questionnaire answers to give to their health care provider during their visits. Combined, the screening and educational materials may prepare patients for a productive conversation with their provider regarding healthy lifestyle choices.

## Figures and Tables

**Table 1:** Demographic and Behavioral Characteristics of Study Subjects, by Their Risk for Diabetes (N = 231), 2003

Characteristic	Total No. (%)	Very Low Risk^a^ No. (%)	Low Risk^b^ No. (%)	High Risk or Diabetes Diagnosis^c^ No. (%)	*P *Value
**Race or ethnicity**					
African American	134 (58.5)	18 (13.4)	70 (52.2)	46 (34.3)	.06
Latina	38 (16.6)	10 (26.3)	19 (50.0)	9 (23.7)	
White	44 (19.2)	15 (34.1)	16 (36.4)	13 (29.6)	
Other	13 (5.7)	4 (30.8)	5 (38.5)	4 (30.8)	
Data missing	2	0	1	1	
**Age, y**					
18-24	56 (24.2)	17 (30.4)	28 (50.0)	11 (19.6)	.01
25-34	75 (32.5)	14 (18.7)	36 (48.0)	25 (33.3)	
35-44	100 (43.3)	16 (16.0)	47 (47.0)	37 (37.0)	
**Education**					
Elementary school	8 (3.5)	1 (12.5)	5 (62.5)	2 (25.0)	.16
Some high school	57 (25.0)	12 (21.1)	25 (43.9)	20 (35.1)	
High school graduate	74 (32.5)	11 (14.9)	41 (55.4)	22 (29.7)	
Some college or technical	68 (29.8)	12 (17.6)	29 (42.6)	27 (39.7)	
College graduate	21 (9.2)	10 (47.6)	9 (42.9)	2 (9.5)	
Data missing	3	1	2	0	
**Days of exercise per week**					
None	53 (22.9)	0	20 (37.7)	33 (62.3)	<.001
1	29 (12.6)	0	11 (37.9)	18 (62.1)	
2	30 (13.0)	0	8 (26.7)	22 (73.3)	
3	40 (17.3)	16 (40.0)	24 (60.0)	0	
4	11 (4.8)	5 (45.5)	6 (54.5)	0	
5 or more	68 (29.4)	26 (38.2)	42 (61.8)	0	
**Servings of fruit or vegetables per day**					
0	16 (6.9)	1 (6.3)	7 (43.8)	8 (50.0)	.01
1-2	130 (56.3)	26 (20.0)	59 (45.4)	45 (34.6)	
3-4	66 (28.6)	12 (18.2)	37 (56.1)	17 (25.8)	
5 or more	19 (8.2)	8 (42.1)	8 (42.1)	3 (15.8)	
**Hours of television watched per day**					
0	8 (3.5)	3 (37.5)	4 (50.0)	1 (12.5)	.08
1	26 (11.3)	9 (34.6)	12 (46.2)	5 (19.2)	
2-3	74 (32.0)	11 (14.9)	36 (48.6)	27 (36.5)	
4-5	69 (29.9)	15 (21.7)	35 (50.7)	19 (27.5)	
6-8	33 (14.3)	6 (18.2)	13 (39.4)	14 (42.4)	
9-12	16 (6.9)	3 (18.8)	8 (50.0)	5 (31.3)	
13 or more	5 (2.2)	0	3 (60.0)	2 (40.0)	

ADA indicates American Diabetes Association. Percentages may not add to 100 because of rounding.

a ADA risk score: 0-2

b ADA risk score: 3-9. Of the 111 in this risk category, 110 had a score of 5-9. They were told they are at low risk now but would be at high risk at age 45 if risk factors do not change.

c ADA risk score: ≥10

**Table 2:** Study Subjects in Each of Five BMI Categories Based on Self-Reported Weight, by BMI Category Based on Scale-Measured Weight (N = 231), 2003

By Self-Reported Weight	By Scale-Measured Weight				

	**Underweight n = 4 (1.7%) No. (%)**	**Healthy Weight n = 58 (25.1%) No. (%)**	**Overweight n = 63 (27.3%) No. (%)**	**Obese n = 84 (36.4%) No. (%)**	**Severely Obese n = 22 (9.5%) No. (%)**
Underweight (BMI <18.5)	3 (75.0)	2 (3.4)	0	0	0
Healthy weight (BMI 18.5-24.9)	1 (25.0)	51 (87.9)	8 (12.7)	0	0
Overweight (BMI 25-29.9)	0	3 (5.2)	50 (79.4)	10 (11.9)	1 (4.6)
Obese (BMI 30-39.9)	0	1 (1.7)	5 (7.9)	70 (83.3)	3 (13.6)
Severely obese (BMI ≥40)	0	1 (1.7)	0	4 (4.8)	18 (81.8)

BMI indicates body mass index. Percentages may not total to 100 because of rounding.

**Table 3:** Study Subjects in Three ADA Risk Scores^a^ Based on Self-Reported Weight, by Scale-Measured Weight, 2003

By Self-Reported Weight	By Scale-Measured Weight		

	**Score 0-2 No. (%)**	**Score 3-9 No. (%)**	**Score ≥10 No. (%)**
**All study subjects (N = 231)**			
Score 0-2	45 (95.7)	6 (5.4)	0
Score 3-9	2 (4.3)	100 (90.1)	4 (5.5)
Score ≥10	0	5 (4.5)	69 (94.5)
Total (all subjects)	47 (20.3)	111 (48.1)	73 (31.6)
**Subjects without a diagnosis of diabetes (n = 214)**			
Score 0-2	44 (97.8)	6 (5.8)	0
Score 3-9	1 (2.2)	94 (90.4)	4 (6.2)
Score ≥10	0	4 (3.8)	61 (93.8)
Total (all subjects without diabetes diagnosis)	45 (21.0)	104 (48.6)	65 (30.4)

ADA indicates American Diabetes Association; BMI body mass index. Percentages may not total 100% because of rounding.

a An ADA risk score of 0-2 is very low risk; 3-9, low risk (those with 5-9 scores were told they are at low risk but likely to be at high risk when aged 45 years or older if risk factors do not change); ≥10, high risk.  **Note**: Of the 111 with *low risk* scores (3-9), 110 had a score of 5-9. They were told they are at low risk now but would be at high risk at age 45 if risk factors do not change.

**Table 4:** Acceptability of Computerized Health Screening to Study Subjects, by Their Diabetes Risk Test Score, 2003

Survey Question	**Total No. (%)**	**ADA Diabetes Risk Test Score^a^**				

		**Very Low RiskScore 0-2 No. (%)**	**Low RiskScore 3-9^b^ No. (%)**	**High RiskScore ≥10 No. (%)**	**Already Have Diabetes No. (%)**	*P* Value^c^
**Is it acceptable to you to answer questions about your health on a computer?**						
Not at all acceptable	20 (8.9)	2 (4.4)	8 (8.2)	6 (9.4)	4 (23.5)	.01
Little to somewhat acceptable	92 (41.1)	18 (40.0)	36 (36.7)	30 (46.9)	8 (47.1)	
Very much to extremely acceptable	112 (50.0)	25 (55.6)	54 (55.1)	28 (43.8)	5 (29.4)	
Data missing	7	0	4	2	1	
**How willing would you be to use a computer again to answer health questions?**						
Not at all acceptable	14 (6.3)	1 (2.2)	5 (5.0)	5 (7.9)	3 (18.8)	.01
Little to somewhat acceptable	58 (25.9)	11 (24.4)	21 (21.0)	21 (33.3)	5 (31.3)	
Very much to extremely acceptable	152 (67.9)	33 (73.3)	74 (74.0)	37 (58.7)	8 (50.0)	
Data missing	7	0	6	1	0	
**Were diet and eating habits discussed with your health care provider?**						
Yes	88 (45.1)	14 (35.9)	44 (51.2)	21 (37.5)	9 (64.3)	.43
No	107 (54.9)	25 (64.1)	42 (48.8)	35 (62.5)	5 (35.7)	
Data missing	36	6	18	9	3	
**If yes, was it helpful?^d^**						
Yes	73 (85.9)	12 (92.3)	34 (81.0)	19 (90.5)	8 (88.9)	.75
No	12 (14.1)	1 (7.7)	8 (19.0)	2 (9.5)	1 (11.1)	
Data missing	3	1	2	0	0	
**If no, would you have liked to discuss?^d^**						
Yes	14 (16.1)	4 (19.0)	6 (18.8)	4 (13.8)	0 (0.0)	.86
No	73 (83.9)	17 (81.0)	26 (81.3)	25 (86.2)	5 (100.0)	
Data missing	20	4	10	6	0	
**Was physical activity discussed with your doctor?**						
Yes	95 (49.0)	20 (50.0)	48 (55.2)	21 (38.9)	6 (46.2)	.26
No	99 (51.0)	20 (50.0)	39 (44.8)	33 (61.1)	7 (53.8)	
Data missing	37	5	17	11	4	
**If yes, was it helpful?^d^**						
Yes	76 (86.4)	15 (88.2)	37 (84.1)	18 (85.7)	6 (100.0)	.96
No	12 (13.6)	2 (11.8)	7 (15.9)	3 (14.3)	0 (0.0)	
Data missing	7	3	4	0	0	
**If no, would you have liked to have discussed?^d^**						
Yes	12 (15.0)	3 (16.7)	5 (17.2)	3 (11.5)	1 (14.3)	.93
No	68 (85.0)	15 (83.3)	24 (82.8)	23 (88.5)	6 (85.7)	
Data missing	19	2	10	7	0	

ADA indicates American Diabetes Association. Percentages may not total to 100 because of rounding.

a Based on scale-measured weight.

b Of the 111 in this risk category, 110 had a score of 5-9. They were told they are at low risk now but would be at high risk at age 45 if risk factors do not change.

c Chi-square test for association (missing data excluded).

d *P* value derived from generalized fisher exact test because of small sample size.
